# Supplementary material for: Efficient replication of influenza D virus in the human airway underscores zoonotic potential
Source: Proc Natl Acad Sci U S A. 2026 Apr 20;123(17):e2530325123. doi: 10.1073/pnas.2530325123 (PMC13123831; doi:10.1073/pnas.2530325123)
Supplement: Supplementary file 1 — Appendix 01 (PDF) [file pnas.2530325123.sapp.pdf]

PNAS

Supporting Information for

Efficient replication of influenza D virus in the human airway underscores zoonotic potential

Christina G. Sanders, Min Liu, Jovanna A. Fusco, Elizabeth M. Ohl, Natalie N. Tarbuck, Emily King, Devra Huey, Thomas P. Fabrizio, Phylip Chen, Amanda R. Panfil, Richard J. Webby, Mark E. Peeples, Andrew S. Bowman, Cody J. Warren

Cody J. Warren
Email: warren.802@osu.edu

This PDF file includes:

Figures S1-S3
Tables S1 and S2

Figures

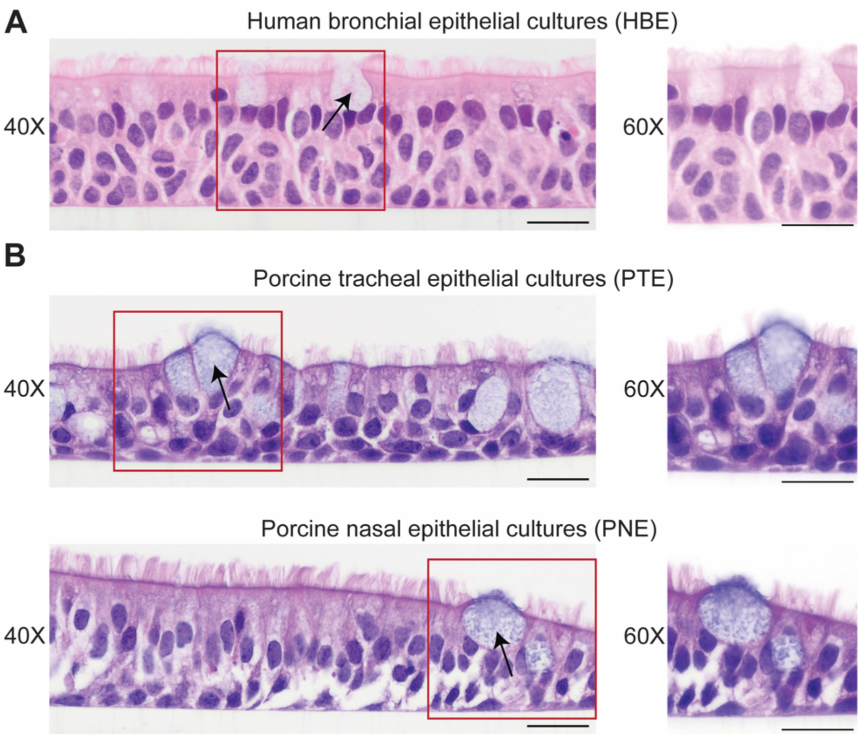

Supplementary Figure 1. Differentiated porcine airway epithelial cultures recapitulate key features of the airway epithelium. Primary porcine nasal epithelial (PNE) and tracheal epithelial (PTE) cultures were grown at the air-liquid interface for three weeks, then paraffin embedded, sectioned, and stained. Representative hematoxylin and eosin (H&E) staining of (**A**) HBE and (**B**) PNE and PTE cultures following differentiation. Images are shown at 40X and 60X magnification, highlighting phenotypic and architectural similarity between porcine and human respiratory culture samples. Arrows denote cell morphologies indicative of mucus-secreting goblet cells. Scale bars = 20 µm. The epithelial layers displayed uniform, elongated cells with distinct borders and minimal variation in cell and nuclear size. Nuclei were round to ovoid, and cilia extended from the apical surface. Goblet cells were interspersed throughout the epithelium, contained basally oriented nuclei, and exhibited lightly stained, mucus-filled cytoplasm, consistent with their expected morphology. Collectively, these features demonstrate strong phenotypic and architectural similarity between porcine and human cultures, supporting their use for meaningful cross-species comparisons during viral infection.

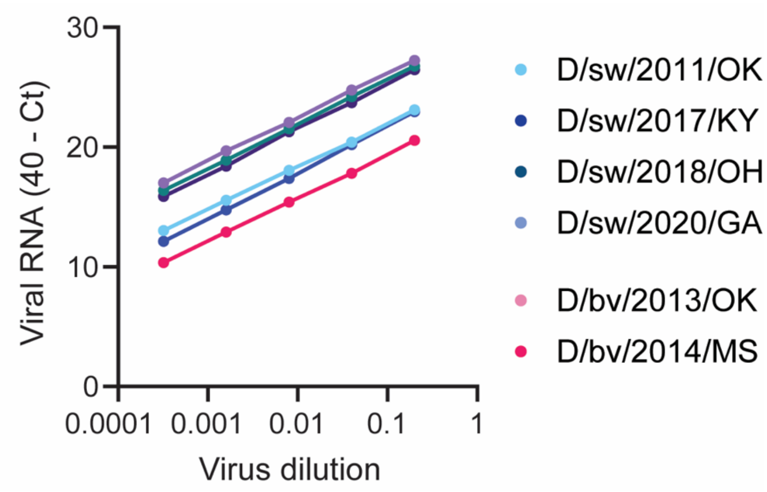

Supplementary Figure 2. Validation of an IDV-specific RT-qPCR assay for high-throughput detection of cell-free viral RNA. Stock viruses were serially diluted 10-fold, followed by viral RNA extraction and rRT-PCR. Linear amplification through a 5-log dilution range is indicative of efficient and sensitive detection of cell-free viral RNA genomes.

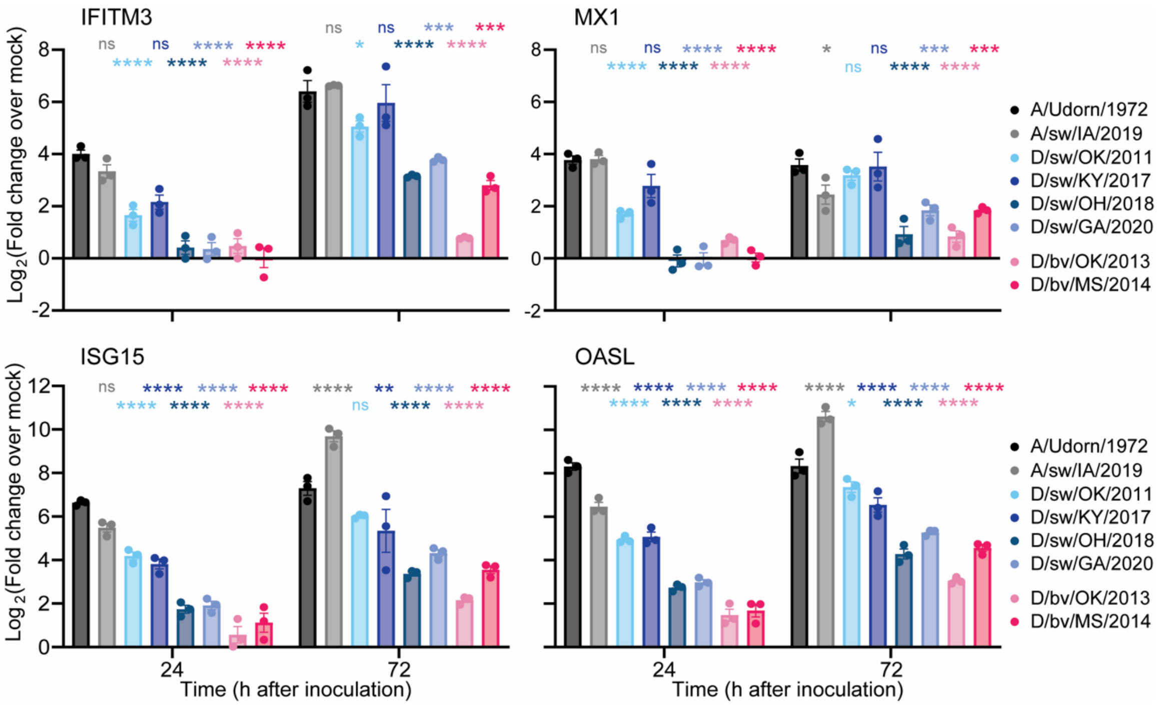

Supplementary Figure 3. IDV induces significantly weaker innate immune responses compared to IAV. Primary airway cultures derived from human (bronchial epithelial, HBE) cells were exposed to IDV at a multiplicity of infection (MOI) of 0.1. Cells were lysed and collected at the indicated time points post inoculation. Total RNA was isolated and interferon stimulated gene (ISG) expression were detected by rRT-PCR. Data show the mean +/- SEM from three independent experiments, with three replicates per experiment. All viruses were compared to wells that were mock infected to calculate fold change (ΔΔCt method, log2 transformed). Two-way ANOVA comparing IDV isolates to IAV control virus (*P<0.5, **P<0.01, ***P<0.001, ****P<0.0001; ns, not significant).

Tables

Supplementary Table 1. Active influenza A surveillance organized by state and

	2017	2018	2019	2020	Total
Arizona		399			399
Colorado			400		400
Georgia		225	395	396	1016
Illinois	399	299	200		898
Indiana	1445	932	905	294	3576
Iowa	799	625	599	1600	3623
Kentucky	759	1309	200	250	2518
Michigan	796	281	324		1401
Mississippi			115		115
Ohio	2854	1973	1948	1602	8377
Oklahoma			900	198	1098
Texas			599		599
West Virginia	20	20			40
Total*	7072	6063	6585	4340	24060

*Numbers indicate total nasal swabs and snout wipes collected by state and year.

Supplementary Table 2. Primer sequences used in this study.

Target	Forward primer (5′ - 3′)	Reverse primer (5′ - 3′)
IDV	TGGATGGAGAGTGCTGCTTC	GCCAATGCTTCCTCCCTGTA
IFNλ1	GGGACCTGAGGCTTCTCC	CCAGGACCTTCAGCGTCA
IFITM3	TTCGCCTACTCCGTGAAGTC	ATCCATAGGCCTGGAAGATCAG
MX1	TATGTGGGTTCCTGGCATCG	AAAGCCTGGCAGCTCTCTAC
ISG15	CAGCGAACTCATCTTTGCCAG	GGACACCTGGAATTCGTTGC
OASL	CCAGCAGTATGTGAAAGCC	AGCCTTCGTCCAACATGA
HPRT1	CATTATGCTGAGGATTTGGAAAGG	CTTGAGCACACAGAGGGCTACA
